# Learning from Instructional Explanations: Effects of Prompts Based on the Active-Constructive-Interactive Framework

**DOI:** 10.1371/journal.pone.0124115

**Published:** 2015-04-08

**Authors:** Julian Roelle, Claudia Müller, Detlev Roelle, Kirsten Berthold

**Affiliations:** 1 Department of Psychology, Bielefeld University, Bielefeld, Germany; 2 Archigymnasium Soest, Soest, Germany; Center for BrainHealth, University of Texas at Dallas, UNITED STATES

## Abstract

Although instructional explanations are commonly provided when learners are introduced to new content, they often fail because they are not integrated into effective learning activities. The recently introduced active-constructive-interactive framework posits an effectiveness hierarchy in which *interactive* learning activities are at the top; these are then followed by *constructive* and *active* learning activities, respectively. Against this background, we combined instructional explanations with different types of prompts that were designed to elicit these learning activities and tested the central predictions of the active-constructive-interactive framework. In Experiment 1, *N* = 83 students were randomly assigned to one of four combinations of instructional explanations and prompts. To test the *active < constructive learning hypothesis*, the learners received either (1) complete explanations and engaging prompts designed to elicit *active* activities or (2) explanations that were reduced by inferences and inference prompts designed to engage learners in *constructing* the withheld information. Furthermore, in order to explore how interactive learning activities can be elicited, we gave the learners who had difficulties in *constructing* the prompted inferences adapted remedial explanations with either (3) unspecific engaging prompts or (4) revision prompts. In support of the *active < constructive learning hypothesis*, we found that the learners who received reduced explanations and inference prompts outperformed the learners who received complete explanations and engaging prompts. Moreover, revision prompts were more effective in eliciting interactive learning activities than engaging prompts. In Experiment 2, *N* = 40 students were randomly assigned to either (1) a reduced explanations and inference prompts or (2) a reduced explanations and inference prompts plus adapted remedial explanations and revision prompts condition. In support of the *constructive < interactive learning hypothesis*, the learners who received adapted remedial explanations and revision prompts as add-ons to reduced explanations and inference prompts acquired more conceptual knowledge.

## Introduction

Written introductory instructional explanations are a common means of introducing learners to new learning content in several media including, for instance, textbooks or computer-based learning environments [1,2]; they include explicit basic information on the new content (e.g., new concepts and principles) that is supposed to be learned. However, although the use of instructional explanations is the rule rather than the exception when learning content is covered for the first time [2], some recent findings have called their effectiveness into question. In their review of instructional explanations literature, Wittwer and Renkl [3] came to the conclusion that instructional explanations often have minimal effects on learning outcomes and posit that these minimal effects could be partially attributed to the fact that instructional explanations are often not integrated into learners’ ongoing cognitive activities. Similarly, Berthold and Renkl [1] argued that mental passivity on part of the learners is an important underlying reason for the frequent failure of instructional explanations. On this basis, they suggested that integrating instructional components designed to elicit learning activities—such as prompts [4,5]—could be a viable approach to overcome this problem. However, although these works highlight the importance of integrating instructional explanations into meaningful learning activities, they do not provide any differentiated suggestions regarding the *type* of learning activities in which learners should be required to engage.

In her *active-constructive-interactive framework*, Chi [6] differentiates between overt *active*, *constructive*, and *interactive* learning activities. She postulates that *active* learning activities are less beneficial than *constructive* learning activities, which, in turn, are less beneficial than *interactive* learning activities [7]. However, when these activity types are applied to the context of learning from introductory instructional explanations, some important issues arise regarding (a) their elicitation and (b) their effects on learning outcomes.

Against this background, in the present studies we combined instructional explanations with different types of prompts that were designed to elicit these three types of learning activities and addressed central hypotheses of the active-constructive-interactive framework. Specifically, in Experiment 1 we (a) addressed the *active < constructive learning hypothesis* and (b) tested two different types of prompts designed to induce interactive learning activities while learners process introductory instructional explanations. In Experiment 2 we employed the type of prompt that had proven to be more effective in eliciting interactive learning activities in Experiment 1 and addressed the *constructive < interactive learning hypothesis*.

## Theoretical Background

In her taxonomy of learning activities, Chi [6] argues that the main characteristic of active learning activities is that learners engage with the learning content without generating information that goes beyond the presented information. For instance, repeating the content of instructional explanations would be an *active* learning activity. In contrast, the main characteristic of constructive learning activities is that learners generate information that not only relates to, but also goes beyond the provided information. Thus, drawing inferences based on the information provided in an instructional explanation would be a *constructive* learning activity. Interactive learning activities, in turn, differ from constructive learning activities in that learners, in addition to being independently constructive, engage in some kind of instructional dialogue in which they both receive and respond to feedback on their constructions [6,7]. For example, if learners who commit errors in the constructive process are provided with remedial explanations that are adapted to their errors (i.e. adapted remedial explanations) and revise them [8], they would be engaging in *interactive* activities.

Chi and colleagues [6,7] not only differentiate active, constructive, and interactive learning with regard to the overt activity type, but also in terms of the underlying cognitive processes that are involved. Under the assumption that these cognitive processes differ in their potential to mediate learning, Chi [6] generates the hypotheses that (a) being constructive fosters learning better than being active and that (b) being interactive, in turn, fosters learning better than being constructive.

### Being Active < Being Constructive

Regarding the differentiation of cognitive processes that relate to active or constructive activities, Fonseca and Chi [7] argue that active activities mainly correspond with *assimilating processes* such as attending to the presented materials, activating relevant prior knowledge, and encoding new information in the context of the relevant activated prior knowledge. These assimilating processes are purported to serve the functions of strengthening and enriching existing knowledge structures [6,7].

Constructive activities also correspond with *assimilating processes* [6,7]. For instance, a learner first has to attend to conceptual information before he/she can construct any inferences that are related to it. Thus, constructive learning activities logically subsume active learning activities. However, the process of constructing inferences also requires *creating processes*. In contrast to mere assimilating processes, these *creating processes* serve the additional function of fostering the coherence and the structure of learners’ knowledge [6,7]. On this basis, Chi derives the hypothesis that being constructive leads to greater learning outcomes than being active. Furthermore, Chi hypothesizes that the critical mediator that leads to the superiority of being constructive is the higher number of self-generated inferences that go beyond the presented information on part of constructive learners. These *active < constructive learning* and *active < constructive via generation hypotheses* are in line with (a) generative theory [9,10], according to which deep understanding requires learners to engage in deep-oriented (constructive) learning activities such as elaborating or integrating new information with prior knowledge, (b) models of text comprehension that highlight the value of mental representations that not only include propositions that are explicitly included in a text, but are also enriched by learner-generated logical inferences and elaborations that go beyond the given information [11–13], and (c) research in cognitive psychology that suggests that self-generated information is better remembered than presented information [14,15].

#### Learning from instructional explanations: Prompting active learning activities < prompting constructive learning activities?

In the field of learning from written introductory instructional explanations, active and constructive activities are often elicited by direct prompting. Recent studies [8,16,17,18] have shown that *engaging prompts* that simply require learners to actively think about the content of instructional explanations (e.g., “Write down your thoughts on the explanation.”) lead novice learners to produce mainly content-repetitions (i.e., a type of active learning activity). Additionally, these studies have also shown that asking specific questions that require learners to generate new information on the basis of provided information (i.e., *inference prompts*) [19–21] is a viable means to foster constructive learning activities when students learn from instructional explanations.

The results of studies that compared both types of prompts are principally in line with the predictions derived from the active-constructive-interactive framework because they consistently found that inference prompts fostered the acquisition of conceptual knowledge when compared to engaging prompts [8,16,22]. However, these results should be taken with caution because in these studies providing introductory explanations together with inference prompts was only compared to providing the *same* introductory explanations together with engaging prompts. These explanations *did not* include the inferences that the inference prompts were designed to elicit. This setting is problematic for two reasons.

Firstly, as responding to inference prompts generates additional content (i.e., the information included in the inferences), the groups that received inference prompts or engaging prompts differed not only in the type of cognitive processes involved (i.e., assimilating and creating processes on part of learners who received inference prompts vs. mere assimilating processes on part of learners who received engaging prompts), but also in the amount of information that was available to them. This put the learners who received engaging prompts at an informational disadvantage because they were not given the opportunity to *attend* to the information the learners who received inference prompts were required to *create*. Therefore, from a theoretical perspective it remains unclear whether the superior performance in acquiring conceptual knowledge in the inference prompt condition was mediated via the *constructive process* (i.e., the generation of inferences) or the *product of the constructive process* (i.e., the generated inferences that went beyond the explanations). In the latter case, providing learners with *complete* instructional explanations that explicitly include the inferences that the inference prompts are designed to elicit in conjunction with engaging prompts should have the same effect as providing *reduced* explanations without the prompted inferences in conjunction with inference prompts.

Secondly, from a practical perspective it is reasonable to assume that instructors who provide their learners with engaging prompts would not give them explanations that lack important inferences. While it is sensible to provide learners with explanations that are reduced by inferences if they are combined with inference prompts that address the withheld inferences, this is not the case if learners merely receive engaging prompts designed to elicit active activities that do not go beyond the provided information. Rather, instructors who use engaging prompts would most likely provide their learners with complete explanations that include *all* the information that needs to be learned. Actually, the latter is an explanation style that is often employed by instructors [15], whereas providing reduced explanations in conjunction with inference prompts is not [1,3]. Thus, from both a theoretical and practical perspective, there is a need to clarify whether providing complete explanations and engaging prompts differs from providing reduced explanations and inference prompts regarding learning outcomes, and if so, why this is the case.

Based on the active-constructive-interactive framework, inference prompts are expected to lead to a higher level of learning from instructional explanations than engaging prompts, even if the available information is balanced. This is due to the higher degree of effectiveness of the creating processes (as opposed to the assimilating processes that occur, for example, while repeating as a response to engaging prompts) that occur while learners are generating inferences in response to inference prompts. Hence, the number of self-generated inferences is likely to serve as a mediator that transmits a potential positive effect of providing reduced explanations and inference prompts as opposed to providing complete explanations and engaging prompts on learning outcomes (*active < constructive via generation hypothesis*).

However, providing reduced explanations in conjunction with inference prompts might also entail a major drawback when compared to providing complete explanations and engaging prompts. Specifically, recent empirical studies that used other learning paradigms suggest that substituting explicitly provided information via prompts designed to elicit the withheld information does not necessarily yield informationally-balanced conditions, but in fact puts constructive learners at a disadvantage. For instance, in a study on animation-based learning, De Koning, Tabbers, Rikers, and Paas [23] found that the learners who had to self-explain an animation generated less than half of the information that was provided to the learners who received explicit explanations that were related to the animation. Furthermore, the authors found that, under these circumstances, being constructive (in this study: generating self-explanations) did not foster learning outcomes. Similarly, results from worked-examples literature [24] and text comprehension research [11] suggest that having learners generate information on their own necessarily involves the risk that they fail to do so correctly. Thus, requiring learners to be constructive not only elicits beneficial inferences, but can also elicit detrimental errors.

Against this background, it is reasonable to assume that substituting inferences that are included in complete instructional explanations with inference prompts might elicit two opposing mediational processes. Compared to providing complete explanations and engaging prompts, reduced explanations and inference prompts might yield not only a *beneficial* mediational effect on the acquisition of conceptual knowledge via the number of self-generated correct inferences, but also a *detrimental* mediational effect via the number of errors (*inconsistent mediation via errors hypothesis*). Therefore, it is unclear whether the *active < constructive learning hypothesis* holds true when active learners are provided with the information that constructive learners have to generate on their own.

### Being Constructive < Being Interactive

The only feature that distinguishes interactive learning activities from constructive learning activities is that the former involves engaging in some sort of instructional dialogue (e.g., with a partner, a tutor, or a system) in which the learners receive *and* respond to feedback on their constructions [6,7]. Correspondingly, in addition to engaging in both assimilating and creating processes that correspond with being constructive, interactive learning activities include the learners’ engagement in *guided creating processes*. These *guided creating processes* are purported to be fundamentally the same as the creating processes involved in constructive learning activities [6,7]. However, as they can draw on information that is not available to learners who solely engage in constructive learning activities on their own, *guided creating processes* go beyond learners’ individual creating processes. Against this background, Chi [6] generates the hypothesis that being interactive should be more beneficial than being constructive (*constructive < interactive learning hypothesis*).

The *constructive < interactive learning hypothesis* is supported by a wealth of studies in the literature on tutoring [25–27] and feedback [28–31], which show that feedback can be a critical component of the learning process. However, it is important to note that, contrary to the widespread argument that the content of the feedback message is the most important aspect of any feedback procedure [32], Chi points out that the type of learning activities learners engage in while processing feedback is crucial as well. Following this line of argumentation, in order to foster learning beyond constructive learning activities, it is not only necessary to provide learners with information that responds to their prior constructive learning activities, but also to ensure that learners actually engage in the targeted interactive learning activities. However, it remains unclear how to best elicit interactive learning activities while learners learn from introductory instructional explanations.

#### How to elicit interactive learning activities in the context of learning from introductory instructional explanations?

In the context of learning from written introductory explanations that are provided in conjunction with inference prompts, eliciting interactive learning activities could be useful if learners have difficulties in generating the prompted inferences on their own. A simple procedure to elicit interactive learning activities could consist of the following three steps: (1) Requiring learners to respond to an inference prompt while processing a reduced introductory explanation. (2) Posing a question that requires the prompted inference. (3) Should the learners have difficulties in answering and/or fail to correctly answer the question, they are given a remedial explanation that includes explicit information on how the prompted inference follows from the respective reduced introductory explanation. As Hattie and Timperley [28] argue that feedback should also prompt active information processing, it could be furthermore combined with an engaging prompt that requires learners to actively think about the remedial explanation.

Nevertheless, it is unclear whether the third step would sufficiently engage learners in interactive learning activities. Sánchez and García-Rodicio [17] found that learners who received remedial explanations and were prompted to express what they were thinking mainly repeated the information included in remedial explanations without explicitly referring to their own misunderstandings. Thus, in terms of the active-constructive-interactive framework [6,7], these learners rarely engaged in interactive learning activities in response to engaging prompts. Additionally, the learners scarcely benefitted from remedial explanations [33–34]. In order to overcome this deficiency, Sánchez and colleagues combined remedial explanations with prompts that were designed to induce revision-oriented learning activities (i.e., revision prompts) [20,21]. In several studies, they consistently found that remedial explanations plus revision prompts fostered both learning outcomes [33,34] and revision-oriented processing of the explanations [17] as compared to providing remedial explanations plus engaging prompts.

However, the findings by Sánchez and colleagues cannot easily be generalized to the broad recommendation that remedial explanations that respond to learners’ prior constructive learning activities should be combined with revision prompts rather than engaging prompts for two reasons: In these studies the remedial explanations were (1) provided after the learners had viewed a computer-based multimedia presentation in which the learners may not have necessarily engaged in constructive learning activities and were (2) designed to remedy *common* misunderstandings relating to the content of the presentation, but *were not* explicitly adapted to the comprehension difficulties of the respective learners. Thus, it is an open question as to whether revision prompts and engaging prompts would also differ in their potential to elicit revision-oriented processing (i.e., interactive learning activities) if learners receive remedial explanations that are *adapted* to their prior *constructive* activities. It is also unclear whether providing constructive learners with adapted remedial explanations and the type of prompts that is more effective in eliciting interactive learning activities would, in accordance with the *constructive < interactive learning hypothesis* [6,7], actually foster learning in comparison to not providing constructive learners with any feedback.

### Hypotheses and Research Questions

Based on these theoretical considerations, we addressed these open questions regarding the *active < constructive* and *constructive < interactive* predictions [6,7] in the context of learning from instructional explanations in two experimental studies. In light of our goal to clarify the effects between engaging prompts that are designed to elicit active activities and inference prompts that are designed to elicit constructive activities, in the first study we were interested in whether learners who receive reduced explanations and inference prompts would acquire more conceptual knowledge than learners who receive complete explanations and engaging prompts (*active < constructive learning hypothesis*). Furthermore, we hypothesized that (a) there would be a positive mediation effect from providing reduced explanations together with inference prompts on the acquisition of conceptual knowledge via the number of self-generated inferences (*active < constructive via generation hypothesis*) and that (b) there would also be a negative mediation effect via the number of errors (*inconsistent mediation via errors hypothesis*). The second goal of the first study was to clarify effects between engaging prompts and revision prompts when they are combined with adapted remedial explanations. Specifically, we were interested in whether engaging prompts and revision prompts would differ in their potential to engage learners in interactive learning activities while they processed the remedial explanations that were adapted to their prior constructive activities.

In the second study, we tested the *constructive < interactive learning hypothesis* in the context of learning from instructional explanations. We wanted to find out whether providing learners with adapted remedial explanations in conjunction with revision prompts (i.e., the type of prompt that had been more effective in eliciting interactive learning activities in the first study) as add-ons to reduced explanations and inference prompts would be superior in fostering learning outcomes in comparison to solely providing learners with reduced explanations and inference prompts.

## Experiment 1

### Materials and Methods

#### Ethics statement

All participants took part on a voluntary basis and their parents gave written informed consent to their participation. All data were collected and analyzed anonymously. The study was conducted in full accordance with the German Psychological Society’s (DGP’s) ethical guidelines (2004, CIII; note that these are based on the APA’s ethical standards) as well as the German Research Foundation’s (DFG’s) ethical standards. According to DFG, psychological studies only need approval from an institutional review board if a study exposes participants to risks that are related to high emotional or physical stress and/or if participants are not informed about the goals and procedures included in the study. As none of these conditions applied to the present study, we did not seek approval from an institutional review board.

In accordance with APA Ethics Code Standard 8.14a, Sharing Research Data for Verification, we agree to make our data available to other qualified professionals for confirmation of analyses and results from the authors on request. All raw data will be retained for a minimum of five years after publication.

#### Sample and design

Eighty-three eighth-grade students of a German high-track secondary school (German: *Gymnasium*, i.e., a college preparatory school) participated in this experiment. The 47 female and 36 male students were between 13 and 15 years old (*M* = 13.69, *SD* = 0.58).

The participants were randomly assigned to one condition of a between-subjects design comprised of four experimental conditions. Specifically, all students received one out of four combinations of written introductory instructional explanations and prompts: They received either (1) complete explanations with engaging prompts designed to elicit active learning activities (*active condition*), (2) reduced explanations with inference prompts designed to elicit constructive learning activities (*constructive condition*), (3) reduced explanations with inference prompts and adapted remedial explanations with engaging prompts that did not explicitly require the learners to engage in interactive learning activities (*interactive/engaging prompts condition*), or (4) reduced explanations with inference prompts and adapted remedial explanations with revision prompts that were explicitly designed to elicit interactive learning activities (*interactive/revision prompts condition*). All instructional explanations were provided in a computer-based learning environment.

#### Computer-based learning environment: Introductory and remedial explanations

The computer-based learning environment consisted of three units and included introductory explanations on 12 basic concepts and principles relating to the structure of atoms that were part of the regular curriculum. We worked in cooperation with the participants’ chemistry teachers in order to design all of the instructional explanations that were included in the learning environment so that they were structurally and explanatorily coherent, meaning there were connective ties between the sentences and that no crucial pieces of background information were left out [20,35,36]. We constructed two versions of each introductory instructional explanation, a complete and a reduced version.

All complete introductory explanations included basic information about concepts or principles related to the topic “atomic structure” and ended with an inference that was based on this basic information. For instance, the complete explanation related to the atomic nucleus included the following information: (1) The number of protons in the core defines the type of atom. (2) The core of an atom can consist of both protons and neutrons. (3) Protons and neutrons have nearly the same weight. (4) The number of protons and neutrons results in the mass number of an atom. The explanation ended with the inference that different types of atoms (e.g., argon and calcium) can have nearly the same mass numbers although they necessarily differ in the number of protons (e.g., argon has 18 protons and calcium has 20 protons) if they differ in the number of neutrons because differences in the number of neutrons can compensate for different numbers of protons (e.g., argon has 22 neutrons and calcium has 20 neutrons; please note that the influence of isotopes is neglected in this explanation, a common didactic simplification in German eighth-grade chemistry lessons). Each complete instructional explanation was provided in conjunction with the engaging prompt “Use the text boxes to write down your thoughts on the explanation.”

In the reduced versions of the explanations, the inferences that were provided at the end of the complete explanations were withheld and the learners were prompted to infer the withheld inferences on their own. For instance, for the explanation relating to the atomic nucleus, the inference prompt was: “How can it be that different types of atoms (e.g., argon, 18 protons and calcium, 20 protons) have nearly the same mass numbers?” The substitution of the inferences that were included in the complete explanations by inference prompts was aimed at balancing the amount of information that was available in the conditions that received complete explanations with engaging prompts and reduced explanations with inference prompts. If the learners who received reduced explanations correctly responded to the inference prompts, they caught up with learners who received complete explanations and engaging prompts. Specifically, the learners who received reduced explanations had to respond to a total of 12 inference prompts while working in the learning environment. Thus, to catch up with learners who received complete explanations regarding the information available, they had to generate 12 inferences on their own.

**Fig 1:**
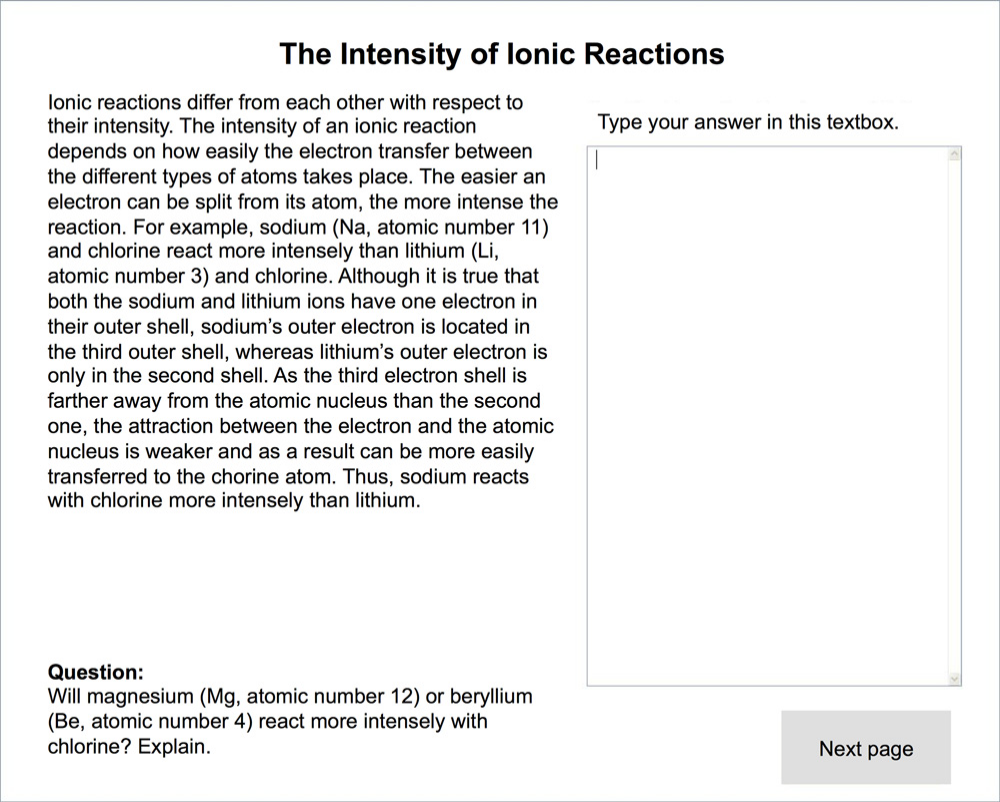
Screenshot of a reduced introductory instructional explanation with inference prompt (translated from German).

Besides the presence or absence of the inferences at the end of the explanations, there were no further differences between the complete and the reduced introductory explanations. All learners were required to type their answers to the prompts into text boxes that were placed next to the explanations (see Fig 1).

After working on an introductory explanation, the learners’ understanding of the given or withheld inference was tested. For this purpose, we adopted the rapid verification approach introduced by Kalyuga [37,38]. The rapid verification approach is a diagnostic method for assessing learners’ domain-specific knowledge structures and has been explicitly designed for the purpose of rapid online application in adaptive computer-based learning environments. Basically, the rapid verification method requires learners to quickly verify whether a suggested step of a problem-solving procedure (e.g., for solving a mathematical problem) is right or wrong [38]. Greater domain specific knowledge should be associated with a greater number of correct verifications. Furthermore, more knowledgeable learners should experience lower levels of difficulty while working on the verification tasks than less knowledgeable learners [38,39].

**Fig 2:**
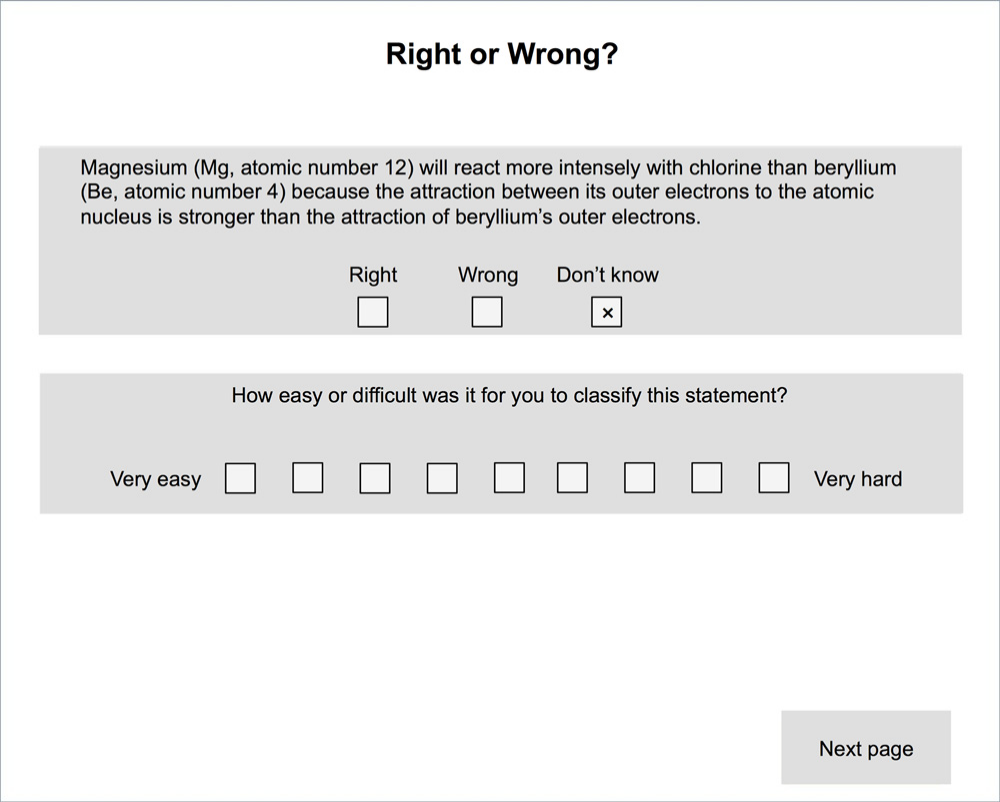
Screenshot of a rapid verification task (translated from German).

In our study, we used a slightly modified version of the rapid verification method [8]. Each introductory instructional explanation was followed by a task that asked the learners to verify an inference. These inferences were predetermined parts of the learning environment and were designed so that they directly related to the inferences at which the inference prompts that were combined with the respective introductory explanation were targeted. For instance, the learners had to verify whether “[…] different types of atoms can only have the same mass numbers if they have a different number of neutrons.” The learners provided their answers by clicking on on-screen buttons (i.e., *right*, *wrong*, and *don’t know*; see Fig 2). They were instructed to click on *don’t know* instead of guessing their responses whenever they were in doubt. Furthermore, the learners had to indicate the difficulty of each verification task on a 9-point rating scale ranging from 1 (*very easy*) to 9 (*very hard*). In case the learners (a) did not correctly verify an inference or (b) rated the difficulty as hard (i.e., a rating of at least 7), the learners in the conditions with adapted remedial explanations received feedback in the form of a remedial explanation that included the correct inference and explained how the inference followed from the previous introductory explanation on the following screen. This adaptation mechanism was the same in both conditions that received remedial explanations.

The learners in the condition that received revision prompts as an add-on to the remedial explanations were given the following prompt together with each remedial explanation: “Does this explanation help you to understand aspects that you did not completely understand before? Please describe which aspects of your prior understanding you revised.” The learners had to type their answers into text boxes. In the condition that received remedial explanations together with engaging prompts, the learners were required to use the text boxes to write down their thoughts on the explanations. On average, the learners in the conditions with adapted remedial explanations received 3.77 (*SD* = 2.43, theoretical max.: 12) adapted remedial explanations (*M*_interactive/revision prompts_ = 3.06, *SD* = 2.30; *M*_interactive/engaging prompts_ = 4.30, *SD* = 2.44). A *t*-test did not yield a statistically significant difference in the number of received remedial explanations between the two conditions with remedial explanations, *t*(38) = 1.63, *p* = .111.

The learners in the conditions without adapted remedial explanations (i.e., the learners who solely received complete explanations and engaging prompts and the learners who solely received reduced explanations and inference prompts) also completed the rapid verification tasks after each introductory explanation. A *t*-test did not show a significant difference in the number of erroneous responses, *t*(41) = 1.46, *p* = .152 (*M*_active_ = 5.52, *SD* = 2.29; *M*_constructive_ = 4.50, *SD* = 2.28). However, irrespective of their responses to the rapid verification tasks, these learners were not provided with any additional explanations.

Please note that due to the small number of participants in each group, the statistical power of the tests regarding the performance on the rapid verification tasks was rather low (i.e., .51 and .36, respectively). Therefore, the results that showed that the respective *t*-tests did not yield statistically significant effects should be interpreted cautiously.

#### Pretest: Assessment of prior conceptual knowledge

A pretest assessed the learners’ prior conceptual knowledge on atomic structure using four open-ended questions (e.g., “How can the number of electrons of an atom be inferred from the number of protons of an atom? Explain your answer.”). The level of comprehension in the learners’ answers was scored using a 6-point rating scale ranging from 1 (*very low level of understanding*) to 6 (*very high level of understanding*). Two independent raters who were blind to the conditions and hypotheses scored the written answers of all participants. Interrater reliability as determined by the intraclass coefficient with measures of absolute agreement was very high for each of the four questions (.91 < ICC < .99). For the later analyses, the scores were averaged into a total score of prior conceptual knowledge (theoretical max.: 6).

#### Prompts responses: Assessment of learning activities

In all conditions, the written responses to the prompts were analyzed. We conducted separate analyses for the introductory and the remedial explanations.

Concerning the written introductory instructional explanations, in order to cover all of the learners’ responses, the text box entries were examined for content segments that corresponded to (1) repetitions, (2) prompted inferences, (3) non-prompted inferences, (4) errors, and (5) monitoring. A content segment was coded as a repetition if the learners wrote down information that was explicitly included in the explanation without adding any new information. Corresponding to our hypothesis that, under informationally-balanced conditions, inference prompts would be better at fostering learning outcomes than engaging prompts because of the higher amount of self-generated information (i.e., constructive learning activities) on part of learners who receive inference prompts, the category *prompted inferences* was explicitly designed to assess the number of inferences the learners showed in response to the inference prompts. However, in order to also assess constructive learning activities that did not relate to inference prompts (note that the learners who received complete explanations could not generate any prompted inference because these were included in the complete explanations), we also coded *non-prompted inferences*. Jointly, these two categories reflect the number of constructive learning activities that all learners engaged in while they processed the introductory explanations. If the content segments included false information (e.g., “Different types of atoms can have the same mass numbers because they have different numbers of electrons.”), they were coded as errors. Segments in which learners indicated that they either understood or did not understand contents of the explanations (e.g., “Now I know the potential ‘ingredients’ of the atomic core” or “I do not understand why there can be different ionization energies within the same shell.”) were coded as monitoring.

Two raters who were blind to the conditions and hypotheses independently coded the responses of all participants. Interrater reliability as determined by Cohen’s kappa was very good (κ = .92). In case of divergence, the coders re-examined the respective cases and made a joint decision. For later analyses, the numbers of repetitions, prompted inferences, non-prompted inferences, errors, and monitoring episodes were summed up over all introductory explanations.

Regarding the adapted remedial explanations, in order to cover all of the learners’ responses, the text box entries were examined for content segments that corresponded to (1) revisions, (2) repetitions, (3) errors, and (4) monitoring. A content segment was coded as a revision if the learners wrote down information that explicitly corresponded with their specific difficulties in responding to the prompts or the respective rapid verification tasks. For instance, if a learner made the aforementioned error (i.e., “Different types of atoms can have the same mass numbers because they have different numbers of electrons.”) in responding to the inference prompt and thus failed to correctly verify the corresponding rapid verification task (i.e., “[…] different types of atoms can only have the same mass numbers if they differ in the number of neutrons.”) and then wrote “Now I understand that the number of electrons is irrelevant for the mass number. The atoms have to differ in the number of neutrons,” it was coded as a revision. The number of revisions reflects the number of interactive learning activities in which the learners engaged. By contrast, if the learners stated information included in the remedial explanations but did not explicitly refer to any of their mistakes, difficulties, or revised aspects (e.g. “Argon and calcium have nearly the same mass numbers because argon has 22 neutrons and calcium has 20 neutrons.”), it was coded as a repetition. Wrong statements were coded as errors. Segments in which learners solely stated that they understood or did not understand contents of the remedial explanations were coded as monitoring.

Two raters who were blind to the conditions and hypotheses independently coded the responses of all participants in the conditions with adapted remedial explanations. Interrater reliability as determined by Cohen’s kappa was very good (κ = .83). In case of divergence, the coders re-examined the respective cases and made a joint decision. For the later analyses, we calculated the average number of revisions, repetitions, errors, and monitoring episodes per received remedial explanation.

#### Posttest: Assessment of learning outcomes

A posttest assessed the learners’ conceptual knowledge on atomic structure after they had processed the written instructional explanations in the computer-based learning environment. The posttest included all four items of the pretest as well as 10 additional open-ended questions. For instance, the learners were asked to explain how the mass number and the number of protons and neutrons of an atom all relate to each other or to explain possible reasons for differences in the ionization energy of different electrons of an atom. These additional questions required a somewhat more advanced understanding of the concepts and principles explained in the learning environment and were thus more difficult than the pretest items. As the learners’ teachers had indicated that the students would know little about these principles and concepts prior to the study, we decided not to include these items in the pretest because this could have frustrated the learners. Note that, due to this decision, the pretest was not strictly parallel to the posttest and did not cover all contents of the learning environment. Therefore, the pretest and the posttest scores were not suitable for a direct comparison.

**Fig 3:**
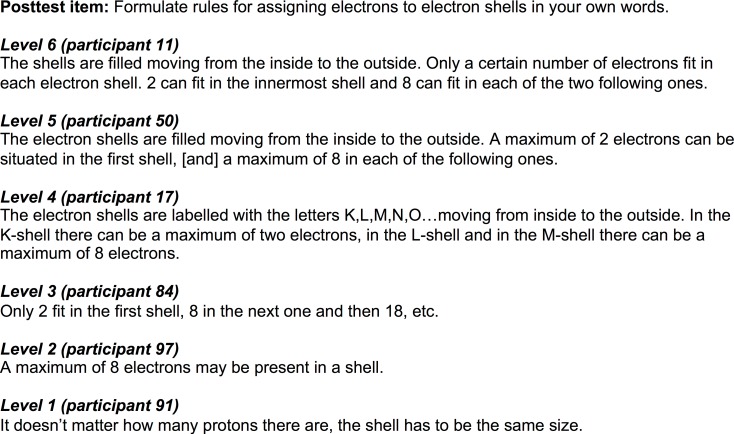
Posttest answers that correspond to the six levels of the rating scale (translated from German).

The level of comprehension in the learners’ answers was scored using a 6-point rating scale ranging from 1 (*very low level of understanding*) to 6 (*very high level of understanding*). Fig 3 provides example answers to one of the questions that relate to these six different levels. Two independent raters who were blind to the conditions and hypotheses scored the written answers of all participants. Interrater reliability as determined by the intraclass coefficient with measures of absolute agreement was very high for each of the questions (.88 < ICC < .99). For the later analyses, we calculated an average score of conceptual knowledge (theoretical max.: 6).

#### Procedure

In group sessions the participants worked individually in front of a computer. First, the participants filled out a demographics questionnaire. Second, they worked on the pretest. Third, all of the learners entered the computer-based learning environment and received a short introduction on how to work in the learning environment before they started the learning phase. Specifically, the participants were told how to use the buttons and the text boxes and that they could work in the learning environment at their individual pace. Furthermore, all learners were informed that they would have to verify statements based on the content of the instructional explanations after each introductory instructional explanation. During the learning phase, the participants worked on either the *complete* or the *reduced* versions of introductory explanations relating to 12 basic concepts and principles of the topic structure of atoms. The participants in the conditions that received reduced explanations and inference prompts had to respond to 12 inference prompts while working in the learning environment. Accordingly, all of the learners had to work on 12 rapid verification tasks. If the learners in the conditions with adapted remedial explanations made errors or had difficulties in responding to a rapid verification task, they received an adapted remedial explanation on the next screen. After completing the learning environment, all participants took the posttest. The experiment lasted approximately 3 hours.

### Results

Table 1 shows the mean scores and standard deviations for each experimental group on the pretest, the posttest, and learning activity measures. To address our hypotheses and research questions, we followed Rosenthal, Rosnow, and Rubin’s [40] recommendations and calculated *a priori* contrasts. In the APA guidelines for the use of statistical methods [41], contrast analysis is a recommended means to address hypotheses and research questions in experimental designs. A major strength of contrast analysis is that it provides the most direct and efficient way to address specific hypotheses or research questions [42]. Hence, except for the pretest scores, all measures were subjected to *a priori* contrasts that corresponded to the hypotheses or research questions. An alpha level of .05 was used for all statistical analyses. To measure effect size, we used *d* qualifying values of approximately 0.20 as small effects, values of approximately 0.50 as medium effects, and values of approximately 0.80 or more as large effects [43].

**Table 1:** Means and (standard deviations) of the pretest, posttest, and learning activity measures in the four experimental conditions of Experiment 1.

	Active condition	Constructive condition	Interactive/engaging prompts condition	Interactive/revisionprompts condition
Pretest	2.21 (0.86)	2.50 (0.92)	2.39 (1.13)	2.36 (0.96)
Posttest	2.07 (0.64)	2.80 (1.22)	2.80 (1.00)	3.41 (1.01)
Introductory explanations:				
Prompted inferences	—	9.40 (4.40)	8.78 (3.81)	10.64 (3.74)
Non-prompted inferences	0.65 (1.77)	0.60 (0.82)	0.48 (0.79)	0.47 (0.62)
Repetitions	13.69 (11.94)	1.25 (2.02)	0.47 (1.27)	0.06 (0.24)
Errors	1.13 (1.51)	4.10 (3.41)	3.60 (2.10)	3.00 (2.18)
Monitoring episodes	0.22 (0.60)	0.00 (0.00)	0.00 (0.00)	0.00 (0.00)
Remedial explanations (per received explanation):				
Revisions	—	—	0.28 (0.30)	1.02 (0.54)
Repetitions	—	—	0.36 (0.42)	0.03 (0.08)
Errors	—	—	0.15 (0.23)	0.04 (0.10)
Monitoring episodes	—	—	0.04 (0.09)	0.09 (0.13)

Regarding the learners’ prior conceptual knowledge, an ANOVA revealed no significant differences between the four experimental groups, *F*(3, 79) = 0.30, *p* = .820. Hence, the experimental groups were comparable in terms of this important learning prerequisite. However, the learners’ prior knowledge was positively correlated with their performance at the rapid verification tasks (*r* = .39, *p* < .001) and the posttest (*r* = .60, *p* < .001). Therefore, we included prior knowledge as a covariate in all subsequent analyses regarding learning outcomes in order to reduce error variance.

Regarding the learners’ posttest performance, an ANCOVA showed a significant main effect of condition, *F*(3, 78) = 8.83, *p* < .001, η^2^ = .25 (large effect). Hence, the four experimental groups differed in their performance on the posttest. This significant overall effect, however, does not directly relate to Experiment 1’s goal of addressing the *active < constructive* prediction. Therefore, we contrasted the active condition to the constructive condition in the following step.

#### Active vs. constructive condition: Effects on learning activities and learning time

As a type of manipulation check, we first analyzed the learning activities of the learners who received complete explanations together with engaging prompts (active condition) or reduced explanations together with inference prompts (constructive condition). We found that the learners in the constructive condition generated a mean of 9.40 (*SD* = 4.41) prompted inferences. This number significantly differed from 12, *t*(19) = 2.64, *p* = .016, *d* = 0.59 (medium effect; one-sample *t*-test). This result suggests that the learners in the constructive condition did not manage to catch up with the learners in the active condition regarding the amount of information that was available. As the learners in the constructive condition had to respond to 12 inference prompts, they (would have) had to generate at least 12 prompted inferences to catch up with the active learners (see Method section). An inspection of the number of errors adds to this picture. We found that the learners in the constructive condition produced more errors than their counterparts in the active condition, *t*(25.42) = 3.59, *p* < .001, *d* = 1.18 (large effect, *t*-test for unequal variances). Thus, although the conditions were designed to be balanced in terms of information available, the numbers of prompted inferences and errors suggest that learners in the constructive condition actually were at a disadvantage.

The principal learning activity of the learners in the active condition was repeating. We found that they produced a mean of 13.69 (*SD* = 11.94) repetitions while processing the complete explanations. Furthermore, we found that they generated a mean of 0.65 (*SD* = 1.77) non-prompted inferences. However, this number did not significantly differ from the number of non-prompted inferences on part of the learners in the constructive condition, *t*(41) = 0.12, *p* = .905. Moreover, we found that they showed a mean of 0.22 (*SD* = 0.60) monitoring episodes while processing the complete explanations.

In light of these differences in the learning activities between the learners in the active and the constructive condition, we also analyzed whether there was a difference in the amount of time the two groups spent on their explanations. We did not find a significant difference, *t*(41) = 0.05, *p* = .958 (*M*_active_ = 44.45, *SD* = 13.22; *M*_constructive_ = 44.64, *SD* = 10.05; in minutes). Hence, although the two groups considerably differed in the type and number of learning activities in which they engaged, there was no difference in learning time. Thus, differences with respect to learning outcomes (see below) cannot simply be attributed to differences in the amount of time spent on the explanations.

#### Active vs. constructive condition: Effects on conceptual knowledge

We were interested in whether the learners in the constructive condition would reach higher levels of conceptual knowledge than the learners in the active condition (*active < constructive learning hypothesis*). We found a significant difference between these two groups, *t*(40) = 2.23, *p* = .015, *d* = 0.70 (medium to large effect, prior knowledge was included as a covariate). The learners in the constructive condition outperformed the learners in the active condition on their posttest scores.

Using only the items that were parallel between the pretest and the posttest, a mixed-repeated measures ANOVA revealed a significant effect of measurement time, *F*(1, 41) = 11.41, *p* = .002, η^2^ = .22 (large effect). The learners generally improved from the pretest to the posttest. Additionally, we found a significant interaction between condition and measurement time, *F*(1, 41) = 5.08, *p* = .030, η^2^ = .11 (medium to large effect). This interaction was due to the fact that the learners in the constructive condition showed higher degrees of improvement than the learners in the active condition. These results are in line with the results on the total posttest performance.

#### Active vs. constructive condition: Mediation analyses

In the *active < constructive via generation hypothesis*, we predicted that a potential superiority of performance of the learners in the constructive condition regarding the acquisition of conceptual knowledge would be mediated via a higher number of self-generated inferences (i.e., constructive learning activities). In the *inconsistent mediation via errors hypothesis*, we also expected that there would nevertheless be a negative mediational effect via a higher number of errors on the part of the constructive learners. To test for the significance of these mediation effects, we followed Preacher and Hayes’ [44] bootstrapping method. In short, these authors point out that the Sobel test [45], which is commonly used to test mediation effects, is based on the unrealistic assumption of the normality of the sampling distribution of the indirect effect. They therefore recommend using a nonparametric resampling procedure, namely computing bootstrap confidence intervals. This procedure means building an empirical approximation of the indirect effect’s sampling distribution through repeatedly resampling the data and estimating the indirect effect thousands of times. Using this method, we generated 95% bias-corrected bootstrap confidence intervals from 5,000 bootstrap samples using the SPSS macro INDIRECT [44].

**Fig 4:**
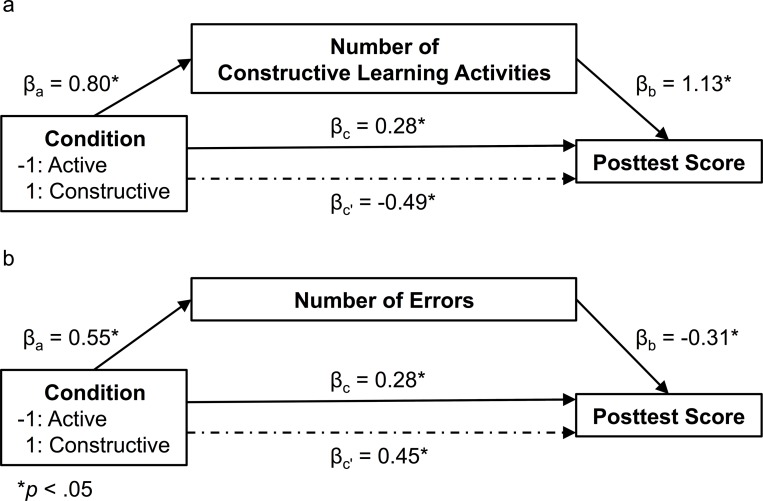
Results of the mediation analyses in Experiment 1.

Regarding the *active < constructive via generation hypothesis*, we found a statistically significant positive indirect effect of the number of constructive learning activities (a×b = 0.78, LCL = 0.47, UCL = 1.14; prior knowledge was included as a covariate; the prompted and non-prompted inferences were aggregated for this analysis). As zero was not in the confidence interval, it can be concluded that there was a positive mediation effect via the number of constructive learning activities on the acquisition of conceptual knowledge (for a path diagram of the mediation results, see Fig 4a).

Concerning the *inconsistent mediation via errors hypothesis*, we found a significant negative indirect effect of the number of errors (a×b = -0.17, LCL = -0.40, UCL = -0.01; prior knowledge was included as a covariate). This result suggests that there also was a negative mediation effect via the number of learner-generated errors on the acquisition of conceptual knowledge (for a path diagram of the mediation results, see Fig 4b).

#### Interactive/engaging prompts vs. interactive/revision prompts condition: Effects on learning activities and learning time

We were interested in whether there would be a difference between engaging prompts and revision prompts in their potential to elicit revisions (i.e., interactive learning activities) while the learners processed the adapted remedial explanations. Note that only the learners who received at least one adapted remedial explanation (*n* = 34) were included in this analysis. We found a statistically significant difference regarding the number of revisions per received adapted remedial explanation, *t*(32) = 5.07, *p* < .001, *d* = 1.87 (large effect). The learners who received adapted remedial explanations and revision prompts (interactive/revision prompts condition) generated more revisions per received remedial explanation than the learners who received adapted remedial explanations and engaging prompts (interactive/engaging prompts condition).

We also analyzed the other learning activities the learners engaged in while processing the adapted remedial explanations. We found that the learners in the interactive/engaging prompts condition produced more repetitions than the learners in the interactive/revision prompts condition, *t*(22.27) = 3.51, *p* = .002, *d* = 1.01 (large effect, *t*-test for unequal variances). However, we found no significant differences between these conditions regarding the number of errors and monitoring episodes, *t*(29.55) = 2.00, *p* = .055 and *t*(18.71) = 1.28, *p* = .214 (*t*-tests for unequal variances), respectively.

In light of these differences with respect to the learning activities, we also analyzed whether the two groups differed in the amount of time they spent on the adapted remedial explanations. We did not find a statistically significant difference, *t*(32) = 0.55, *p* = .589 (*M*_interactive/engaging prompts_ = 9.21, *SD* = 5.19; *M*_interactive/revision prompts_ = 10.22, *SD* = 5.31; in minutes).

#### Interactive/engaging prompts vs. interactive/revision prompts condition: Effects on conceptual knowledge

For exploratory purposes, we also analyzed whether, according to the *constructive < interactive learning hypothesis* [6,7], the two interactive conditions outperformed the constructive condition regarding the acquisition of conceptual knowledge. However, as these analyses are based on non-orthogonal contrasts, they should be interpreted cautiously. We did not find a significant difference between the constructive and the interactive/engaging prompts condition, *t*(40) = 0.31, *p* = .379. In contrast, we did find a significant difference between the constructive and the interactive/revision prompts condition, *t*(34) = 2.39, *p* = .011, *d* = 0.82 (large effect). The learners in the interactive/revision prompts condition reached higher posttest scores. We also found that the learners in the interactive/revision prompts condition had spent more time in the learning environment than the learners in the constructive condition, *t*(35) = 2.18, *p* = .036, *d* = 0.74 (medium to large effect). However, learning time was not correlated with the posttest scores, *r* = -.03, *p* = .860. Nevertheless, we tested whether learning time served as a mediator of the superior performance of the learners in the interactive/revision prompts condition on the posttest scores. We did not find a statistically significant mediation effect via learning time (a×b = 0.02, LCL = -0.06, UCL = 0.21; prior knowledge was included as a covariate). Specifically, as zero was in the confidence interval, it can be concluded that the superiority of the learners in the interactive/revision prompts condition regarding posttest scores was not simply due to more learning time.

### Discussion

The main findings from Experiment 1 were: (a) In support of the *active < constructive learning hypothesis*, inference prompts were more beneficial for the acquisition of conceptual knowledge than engaging prompts even though the latter were combined with complete explanations that explicitly included the inferences that were addressed by the inference prompts. Furthermore, in line with the *active < constructive via generation hypothesis*, the process of learners generating information on their own mediated the superiority of reduced explanations and inference prompts over complete explanations and engaging prompts. (b) In support of the *inconsistent mediation via errors hypothesis*, providing reduced explanations and inference prompts rather than complete explanations and engaging prompts yielded inconsistent mediational effects on the acquisition of conceptual knowledge because it not only led to an increase in the number of beneficial self-generated inferences, but also in the number of detrimental errors. (c) Revision prompts were better at fostering the elicitation of interactive learning activities while processing adapted remedial explanations than engaging prompts. Furthermore, adapted remedial explanations were only a beneficial add-on to reduced explanations and inference prompts when they were combined with revision prompts.

#### Active < constructive: Complete explanations and engaging prompts < reduced explanations and inference prompts

The pattern of results can be interpreted as follows: In line with previous findings [8,16,22], engaging prompts mainly caused the learners to repeat the informational content included in the introductory explanations (an active learning activity), whereas inference prompts mainly led the learners to generate prompted inferences (a constructive learning activity). Furthermore, in support of the *active < constructive learning hypothesis* [6,7] and in line with the results of previous studies that had compared inference prompts and engaging prompts [8,16,22], we found that the learners in the constructive condition acquired more conceptual knowledge than the learners in the active condition.

However, it is important to note that in the present study, reduced explanations and inference prompts were more effective even though, in contrast to previous studies [8,16,22], the learners who received engaging prompts were provided with *complete* explanations that explicitly included all of the inferences that the learners who received inference prompts were prompted to generate on their own. Notably, this compensation mechanism actually yielded an informational disadvantage for the learners in the constructive condition. Similar to results reported by De Koning et al. [23], the learners in the constructive condition did not manage to generate correct inferences in response to every inference prompt. Furthermore, they produced higher numbers of errors. Hence, they did not catch up with the learners in the active condition in terms of information that was available to them. Nevertheless, in line with the *active < constructive via generation hypothesis*, the results of our mediation analyses suggest that the higher number of constructive learning activities on part of the learners in the constructive condition caused the inference prompts’ beneficial effect on the level of conceptual knowledge. As the learners in the active condition were explicitly given all prompted inferences, these results, rather than reflecting an informational advantage on part of the constructive learners, reflect the superiority of engaging in constructive rather than engaging in active learning activities. Thus, complementing previous studies that compared engaging prompts and inference prompts [8,16,22], Experiment 1 shows that inference prompts may not only be beneficial *supplements* to written introductory explanations, but also beneficial *substitutes for information* provided in the introductory explanations.

This *active < constructive learning conclusion*, however, is challenged by two restrictions. First, it should be noted that the learners in the two conditions not only engaged in (active or constructive) learning activities while they worked in the learning environment, but also took the rapid verification tasks. As working on these tasks required retrieval from memory, these tasks have to be viewed as an intervention that affects learning rather than as a form of neutral assessment [46]. Hence, the learners’ posttest performance might have been influenced by both their respective learning activities and the rapid verification tasks. The rapid verification tasks, however, were the same for all participants (see Method section). Therefore, it is unlikely that the significant difference regarding posttest performance was due to this factor.

The second restriction relates to the negative effect of learner-generated errors. Although the learners in the constructive condition ultimately acquired more conceptual knowledge, their informational disadvantage appeared to have significant negative consequences. In line with the *inconsistent mediation via errors hypothesis*, we found that the number of errors entailed a detrimental effect on the acquisition of conceptual knowledge. Hence, although combining reduced explanations with inference prompts fostered the generation of beneficial inferences, it also raised the risk of making detrimental errors and thus partly counteracted the beneficial effect of requiring learners to be constructive. Although this double-edged effect of providing learners with reduced explanations and inference prompts needs to be replicated in further studies, the results of this study suggest that the *active < constructive learning hypothesis* does not hold true in all cases because there might be settings in which the negative effect of learner-generated errors outweighs the positive effect of learner-generated inferences. This tentative assumption, however, needs to be addressed in future studies.

Besides pointing to a restriction of the *active < constructive learning conclusion*, the increased risk of making detrimental errors on part of constructive learners also points to a potential to optimize the procedure of providing learners with reduced explanations in conjunction with inference prompts. Specifically, the result that the learners did not manage to correctly generate all of the prompted inferences suggests that remedial explanations that are adapted to learners’ difficulties or errors might be a beneficial add-on to this procedure.

#### Eliciting interactive learning activities while learners process adapted remedial explanations: Engaging prompts < revision prompts

Regarding the comparison of providing learners with adapted remedial explanations in conjunction with either *engaging prompts* (interactive/engaging prompts condition) or *revision prompts* (interactive/revision prompts condition) in case they had difficulties in generating the prompted inferences, our results can be interpreted as follows: Adapted remedial explanations with revision prompts as add-ons to reduced explanations and inference prompts were better at fostering the revision of errors. Whereas the learners in the interactive/revision prompts condition showed about 1.02 revisions per received remedial explanation on average, the learners in the interactive/engaging prompts condition merely showed 0.28 revisions per remedial explanation (see Table 1). Hence, in terms of the active-constructive-interactive framework [6,7], revision prompts were more effective than engaging prompts in eliciting interactive learning activities.

These results complement previous findings concerning the effects of providing written remedial explanations together with engaging prompts or revision prompts in computer-based learning environments [17,33,34]. In these studies, remedial explanations were provided after the learners had viewed a computer-based multimedia presentation. However, as learning activities were not examined while the learners were watching the presentation, the extent to which the explanations responded to active or constructive learning activities is unclear. Additionally, in these studies the remedial explanations were adapted to common misunderstandings that learners tend to have in general rather than to the specific difficulties of each learner. The researchers constantly found there was little benefit from remedial explanations unless they were combined with revision prompts. In light of our findings, we can add the point that even when the remedial explanations were (a) provided directly after the learners had engaged in constructive learning activities and were (b) adapted to the learners’ specific comprehension difficulties, engaging prompts barely elicited revision-oriented processing (i.e., interactive learning activities) and, consequently, had minimal effects on learning outcomes.

Our results also complement recent findings regarding the effects of feedback. In their study on explanatory feedback, Butler et al. [32] argued that the content of the feedback message is the most important aspect of any feedback procedure. In our study, the conditions that received remedial explanations were designed to be balanced in terms of the information available. The content of the feedback messages (i.e., the content of the remedial explanations) and the adaptation mechanism was the same in these two conditions. However, our exploratory analyses regarding learning outcomes showed that only the learners in the interactive/revision prompts condition outperformed the learners in the constructive condition, whereas the learners in the interactive/engaging prompts condition did not. One explanation for this pattern of results is that the number of interactive learning activities on part of the learners in the interactive/engaging prompts condition was too low. As these learners merely produced an average of 0.28 revisions per received remedial explanation, they scarcely went beyond their prior constructive learning activities. Consequently, although they received remedial feedback, they did not acquire more conceptual knowledge than the learners in the constructive condition. Hence, in line with Chi’s [6] argumentation, our results indicate that, in order to support learning that goes beyond learners’ self-generated constructions, both the content of the provided feedback and the activities in which learners engage while they process it should be optimized by instructors.

## Experiment 2

One restriction of our analyses regarding the *constructive < interactive learning hypothesis* in Experiment 1 is that they were exploratory and merely based on non-orthogonal contrasts. Therefore, in order to strengthen and generalize the finding that adapted remedial explanations that are provided in conjunction with revision prompts are a beneficial add-on to reduced explanations that are provided in conjunction with inference prompts, we sought to replicate this finding in a second experiment.

### Materials and Methods

#### Ethics statement

We followed the same procedure and agreed to the same standards used in Experiment 1.

#### Sample and design

Forty eleventh-grade students of a German high-track secondary school (German: *Gymnasium*) participated in this experiment. The 28 female and 12 male students were between 16 and 19 years old (*M* = 17.00, *SD* = 0.93).

The participants were randomly assigned to one condition of a between-subjects design with two experimental conditions. The learners received either (1) reduced introductory explanations with inference prompts designed to elicit constructive learning activities (*constructive condition*), or (2) reduced explanations with inference prompts and adapted remedial explanations with revision prompts designed to elicit interactive learning activities (*interactive/revision prompts condition*). All instructional explanations were provided in a computer-based learning environment.

#### Computer-based learning environment: Introductory and remedial explanations

The learning environment consisted of introductory explanations on 12 basic concepts and principles in the domain of biology that were part of the regular curriculum (specific topics: Mendel’s laws of inheritance and basic principles of human genetics). All of the instructional explanations that were included in the learning environment were designed so that they were structurally and explanatorily coherent, meaning there were connective ties between the sentences and that no crucial pieces of background information were left out [20,35,36].

All of the reduced introductory explanations included basic information about concepts or principles related to Mendel’s laws of inheritance or human genetics such as the concept of diploidy or the principle of uniformity. Each reduced introductory explanation was presented together with an inference prompt that was designed to elicit an inference that was based on the central content of the introductory explanation. For instance, the introductory explanation on the principle of uniformity was accompanied by the following inference prompt: “According to the principle of uniformity, which possible combinations of hereditary traits can the next generation inherit? Please include genotypes and phenotypes in your explanation.” The learners had to type their answers to the inference prompts into text boxes that were placed under the introductory explanations on the computer screen.

We used the same adaptation mechanism as the one in Experiment 1. Hence, each introductory instructional explanation was followed by a task that required the learners to verify an inference that directly related to the inference that the inference prompt they had worked on before was targeted at. For instance, after responding to the aforementioned inference prompt, the learners had to verify the following inference: “According to the principle of uniformity, a successive generation always results in a homozygous filial generation.” On average, the learners in the interactive/revision prompts condition received 2.94 (*SD* = 2.25, theoretical max.: 12) adapted remedial explanations. In order to prevent confounding through retrieval-based learning while responding to the rapid verification tasks [46], the learners in the condition without adapted remedial explanations also completed these tasks after each introductory explanation (*M*_errors_ = 4.13, *SD* = 2.63). A *t*-test did not reveal a statistically significant difference in performance on these tasks between the two groups, *t*(38) = 1.50, *p* = .142. However, due to the small number of participants in each group, the statistical power of this test was rather low (i.e., .46). Therefore, the result that the *t*-test did not yield a statistically significant effect should be interpreted cautiously.

#### Pretest: Assessment of prior conceptual knowledge

We administered a pretest with four open-ended questions to assess the learners’ prior conceptual about the topics of the learning environment. For instance, in one of the questions the learners were asked to explain the concept of diploidy or the principle of uniformity. Based on a scoring protocol, the learners’ answers were examined for correct arguments. Two independent raters who were blind to the conditions and hypotheses scored the written answers of all learners. Interrater reliability as determined by the intraclass coefficient with measures of absolute agreement was very high for each of the four questions (.89 < ICC < 1). For the later analyses, the scores were aggregated into a total score of prior conceptual knowledge (theoretical max.: 15).

#### Prompts responses: Assessment of learning activities

Using the same coding schemes as the ones used in Experiment 1, the written responses to the prompts were examined for different types of content segments. As in Experiment 1, we conducted separate analyses for the introductory and the remedial explanations. In our analyses of the introductory explanations, the category “monitoring” was ultimately left out because we did not find any monitoring episodes. Regarding the adapted remedial explanations, the category “repetitions” was ultimately left out because we did not find any repetitions. Interrater reliability as determined by Cohen’s kappa was very high for both the analyses regarding the introductory explanations and the remedial explanations (both κ > .80).

#### Posttest: Assessment of learning outcomes

A posttest with eight open-ended questions assessed the learners’ conceptual knowledge about the topics Mendel’s laws and human genetics. The posttest included all four pretest items as well as four additional open-ended questions. For instance, the learners were asked to explain the central attributes of hereditary transmission that follows the law of segregation or to explain the law of independent assortment. Based on a scoring scheme, the learners’ answers were examined for correct arguments. Two independent raters who were blind to the conditions and hypotheses scored the written answers of all participants. Interrater reliability as determined by the intraclass coefficient with measures of absolute agreement was very high for each of the eight questions (.91 < ICC < .98). For the later analyses, the scores were aggregated into a total score of conceptual knowledge (theoretical max.: 31).

#### Procedure

The procedure was identical to the one used in Experiment 1. The experiment lasted approximately 2 hours.

### Results

Table 2 shows the mean scores and standard deviations for both experimental groups on the pretest, the posttest, and learning activity measures. An alpha level of .05 was used for all statistical analyses. To measure effect size, we used *d* qualifying values of approximately 0.20 as small effects, values of approximately 0.50 as medium effects, and values of approximately 0.80 or bigger as large effects [43].

**Table 2:** Means and (standard deviations) of the pretest, posttest, and learning activity measures in the two experimental conditions of Experiment 2.

	Constructive condition	Interactive/revision prompts condition
Pretest	1.32 (1.86)	2.02 (1.86)
Posttest	8.49 (5.77)	12.84 (6.83)
Introductory explanations:		
Prompted inferences	6.50 (3.97)	7.59 (2.87)
Non-prompted inferences	0.05 (0.21)	0.23 (0.75)
Repetitions	3.18 (1.89)	3.82 (1.94)
Errors	2.32 (1.49)	2.82 (1.51)
Remedial explanations (per received explanation):		
Revisions	—	0.90 (0.61)
Errors	—	0.01 (0.05)
Monitoring episodes	—	0.07 (0.18)

Regarding the learners’ prior conceptual knowledge, we found no significant difference between the two experimental groups, *t*(38) = 1.26, *p* = .214. Hence, the experimental groups were comparable in terms of this important learning prerequisite. However, the learners’ prior knowledge was positively correlated with their performance on the rapid verification tasks (*r* = .73, *p* < .001) and the posttest (*r* = .48, *p* = .002). Therefore, we included prior knowledge as a covariate in all subsequent analyses regarding learning outcomes to reduce error variance.

#### Effects on learning activities and learning time

As a type of manipulation check, we first analyzed the learning activities of the learners in the constructive and the interactive/revision prompts condition. Regarding the introductory explanations, we found that the learners generated a mean of 6.97 (*SD* = 3.53) prompted inferences in response to the 12 inference prompts (due to technical problems, the text box entries of one participant were lost). There was no significant difference between the two groups, *t*(37) = 0.95, *p* = .347. Furthermore, we found that the learners produced 0.13 (*SD* = 0.52) non-prompted inferences, 3.46 (*SD* = 1.92) repetitions, and 2.53 (*SD* = 1.50) errors while they processed the introductory explanations. As for the number of prompted inferences, we did not find any significant differences between the two groups regarding these learning activities, 1.03 < *t*(37) < 1.14.

With respect to the adapted remedial explanations, we found that the learners in the interactive/revision prompts group showed a mean of 0.90 (*SD* = 0.61) revisions per received remedial explanations (only learners who had received at least one adapted remedial explanation were included in this analysis, *n* = 15). Furthermore, we found that on average they showed 0.07 (*SD* = 0.18) monitoring episodes and 0.01 errors (*SD* = 0.05) per received remedial explanation.

As the learners in the interactive/revision prompts group generally received more (remedial) explanations than the learners in the constructive condition, we also analyzed whether the two groups differed with regards to the amount of time spent in the learning environment. We found a marginally significant difference, *t*(38) = 1.86, *p* = .070, *d* = 0.61 (medium effect). The learners in the interactive/revision prompts condition spent more time in the learning environment than the learners in the constructive condition (*M*_constructive_ = 31.38, *SD* = 13.91; *M*_interactive/revision prompts_ = 39.02, *SD* = 11.13; in minutes).

#### Effects on conceptual knowledge

We were interested in whether the learners in the interactive/revision prompts condition would acquire more conceptual knowledge than the learners in the constructive condition (*constructive < interactive learning hypothesis*). We found a statistically significant effect, *t*(40) = 1.74, *p* = .045, *d* = 0.57 (medium effect, prior knowledge was included as a covariate). The learners in the interactive/revision prompts condition had higher scores on the posttest than the learners in the constructive condition.

Using only the items that were parallel between the pretest and the posttest, a mixed-repeated measures ANOVA revealed a significant effect of measurement time, *F*(1, 38) = 16.67, *p* < .001, η^2^ = .31 (large effect). The learners generally improved from the pretest to the posttest. Additionally, we found a significant interaction between condition and measurement time, *F*(1, 38) = 4.46, *p* = .041, η^2^ = .11 (medium to large effect). This interaction was due to the fact that the learners in the interactive/revision prompts condition showed higher degrees of improvement than the learners in the constructive condition. These results are in line with the results regarding the total posttest performance.

As the learners in the interactive/revision prompts condition spent more time in the learning environment than learners in the constructive condition, we also analyzed the relation between posttest scores and learning time. We found that learning time was positively correlated with posttest scores, *r* = .53, *p* < 001. Therefore, in the following step we tested whether learning time served as a mediator of the superiority of the learners in the interactive/revision prompts condition regarding posttest scores. However, we did not find a significant mediation effect (a×b = -1.11, LCL = -3.18, UCL = 0.10; prior knowledge was included as a covariate). As zero was in the confidence interval, it can be concluded that the superiority of the learners in the interactive/revision prompts condition on the posttest scores was not due to spending more time in the learning environment.

### Discussion

In sum, the results of Experiment 2 strengthen our finding of Experiment 1, which showed that adapted remedial explanations that were combined with revision prompts are a beneficial add-on to reduced explanations and inference prompts. Using a second sample of high school students and different learning materials, similar to Experiment 1 we found that revision prompts caused the learners to revise their errors (i.e. interactive learning activities) to a substantial degree while they processed the adapted remedial explanations. Furthermore, in support of Chi’s [6] *constructive < interactive learning hypothesis*, we found that the learners in the interactive/revision prompts condition acquired more conceptual knowledge than the learners in the constructive condition.

## General Discussion

In summary, the present research (a) shows how active, constructive, and interactive learning activities can be elicited while learners are learning from written introductory instructional explanations through prompting and (b) provides experimental evidence concerning the main hypotheses behind the active-constructive-interactive framework [6,7] in this learning paradigm. Regarding the elicitation of the three types of learning activities, we found that engaging prompts mainly elicited active learning activities such as repeating, but ended up being of little use for the purpose of eliciting constructive or interactive learning activities. By contrast, in line with previous studies [8,16,22], inference prompts proved to be an effective means to elicit constructive learning activities. However, our results also point to some limitations of providing learners with inference prompts. In neither experiment did the inference prompts actually elicit the targeted number of constructive learning activities. In Experiment 1 the learners in the constructive condition managed to generate about 9.40 correct inferences in response to 12 inference prompts, and in Experiment 2 the ratio of inferences per inference prompt was even lower. These findings support the argumentation put forward in the active-constructive-interactive framework [6,7], which states that, after engaging in creating processes on their own, learners should be encouraged to engage in guided creating processes in which learners can build on feedback from a tutor or a system.

In the context of learning from adapted remedial explanations, our studies suggest that revision prompts are an effective means to engage learners in guided creating processes (i.e., interactive learning activities). In Experiment 2, revision prompts elicited about 0.90 revisions per received remedial explanation, and in Experiment 1 the ratio was even higher. Furthermore, in support of the *constructive < interactive learning hypothesis* [6,7] we found that the learners in the interactive/revision prompts conditions acquired more conceptual knowledge than learners in the constructive conditions in both experiments. Our results suggest that this superiority of the interactive/revision prompts conditions may *not* be simply attributed to a higher amount of learning time on part of the interactive learners. Although the interactive learners spent more time in the learning environment than the learners in the constructive conditions in both studies, mediation analyses showed that learning time did not serve as a mediator of the superiority of the interactive learners regarding posttest scores in either case. Additionally, the results in Experiment 1 showed that learners in the interactive/engaging prompts condition had spent nearly the same amount of time in the learning environment as the learners in the interactive/revision prompts condition. In spite of this, they did not outperform the learners in the constructive condition, whereas the learners in the interactive/revision prompts condition did. This suggests that it was the engagement in interactive learning activities (which of course needed time) and not the higher learning time per se that tipped the scale in favor of the learners in the interactive/revision prompts conditions.

One restriction regarding our findings with respect to the use of interactive learning activities is that we implemented a relatively *parsimonious* version of interactive learning activities. If the learners in the interactive conditions in both experiments experienced difficulties or even failed to verify inferences that closely related to the inference prompts they had responded to before, they received remedial explanations that highlighted how the inferences followed from the introductory explanations. Hence, the adapted remedial explanations provided the learners with the opportunity to surpass their (erroneous) creating processes and thus served as the basis for guided creating processes. Based on this interpretation, this setting met the *basic* requirements of interactive learning settings as defined by Chi [6]. Furthermore, the results show that the learners in the interactive conditions in both experiments engaged in interactive learning activities and used the adapted remedial explanations to revise their errors. Nevertheless, it is reasonable to assume that the setting used in the present studies was not suitable to exploit the full potential of interactive learning activities. For instance, Chi argues that in dialogues with an instructor or a system, corrective feedback is often followed by an extended dialogue on the respective issues. Although the learners in both experiments of the interactive/revision prompts condition rarely committed any errors and generated substantial numbers of revisions while they processed the remedial explanations (see Table 1 and Table 2), the amount of feedback the participants received in the present studies was rather limited. Had the participants the opportunity to engage in more extended dialogues, they might have acquired more conceptual knowledge as a result of more guided processing through interaction. Therefore, our results should not be interpreted as a reflection of the full potential of interactive learning activities in the context of learning from introductory explanations. However, it could also be argued that our studies show that even relatively simple versions of interactive learning activities entail a higher potential to foster learning than constructive activities that learners can engage in on their own. Evidently, despite the relative parsimonious implementation of interactive learning activities, we found medium to large effects between the constructive and the interactive/revision prompts conditions in both of our experiments.

### General Limitations

A general restriction of the present studies is the modality of the explanations. In both of our experiments all of the instructional explanations were provided in a written form. Thus, it is unclear whether our findings could also be generalized to settings that include spoken introductory instructional explanations. In settings that include spoken explanations, one should bear in mind that the information included in the explanations is transient. A major consequence of transient information is that it generally raises working memory load [47,48]. Given that the learning activities elicited by engaging prompts, inference prompts, or revision prompts also place different demands on cognitive load, it is conceivable that the effects of the different types of prompts might change in the spoken mode. For instance, because inference prompts require one to engage in creating processes, they could entail higher demands on working memory load than engaging prompts. Consequently, the combination of spoken explanations and inference prompts is more likely to lead to a cognitive overload than the combination of spoken explanations and engaging prompts and could therefore counteract the findings reported in Experiment 1. In the field of animation-based learning findings by De Koning et al. [23] suggest that providing complete explanations might work just as well as requiring learners to be constructive. However, in this study the constructive learners were not explicitly prompted to generate the information that was included in the complete explanations. Therefore, the effect cannot only be attributed to the different types of activities in which the learners engaged. Nevertheless, the results of De Koning et al.’s study suggest that exploring potential moderations of the effects of engaging prompts, inference prompts, and revision prompts by the modality in which introductory instructional explanations are presented might be a fruitful line of further research.

A second general restriction refers to the design of our studies. We strived to clarify whether engaging prompts and inference prompts would differ in their potential to mediate learning under informationally-balanced conditions as suggested by the active-constructive-interactive framework and whether revision prompts and engaging prompts would differ in their potential to elicit interactive learning activities when they are combined with remedial explanations that are adapted to learners’ difficulties. Furthermore, we were interested in whether adapted remedial explanations in conjunction with revision prompts would be a beneficial add-on to reduced explanations and inference prompts. Against this background, in Experiment 1 we decided to combine reduced explanations with inference prompts and complete explanations with engaging prompts, but not to use a complete factorial design that included all possible combinations. As a consequence, this study cannot answer whether providing complete explanations in conjunction with engaging prompts is more beneficial than providing reduced explanations and engaging prompts [18] or whether providing remedial explanations would support the learners who received complete explanations and engaging prompts in catching up with the learners who received reduced explanations and inference prompts. Nevertheless, regarding the latter question, both our findings as well as those reported by Sánchez and colleagues [17,33,34] jointly support the assumption that remedial explanations and revision prompts foster learning, regardless of the type of prior learning activities (e.g., *active* repeating or *constructive* inferring). However, future studies should challenge this tentative assumption by explicitly comparing the effects of providing remedial explanations after learners have engaged in different types of learning activities.

### Implications for Instructional Design

Despite the aforementioned restrictions and implications for further research, our studies imply the following conclusions with respect to instructional design: When instructors plan to introduce learners to new principles and concepts in computer-based learning environments via written instructional explanations, they should consider substituting inferences that can be inferred on the basis of the remaining information with inference prompts instead of providing learners with complete explanations that include all of the information that needs to be learned in conjunction with engaging prompts. The results of our experiments showed that providing reduced explanations and inference prompts is better at fostering both constructive learning activities and the acquisition of conceptual knowledge than providing complete explanations and engaging prompts. As a caveat, instructors should be aware that eliciting constructive learning activities also increases the risk that learners fail to engage in creating processes successfully, which can counteract the superiority of providing reduced explanations and inference prompts instead of complete explanations and engaging prompts.

In considering potential remedies with respect to learner-generated errors, our findings imply that instructors should not assume that learners sufficiently build on remedial instructional explanations that provide feedback to their comprehension difficulties in all situations. The results of Experiment 1 showed that adapted remedial explanations only contributed to interactive learning activities when they were combined with revision prompts. Hence, instructors should consider combining the procedures of providing reduced explanations together with inference prompts and adapted remedial explanations together with revision prompts in computer-based learning environments that are designed to introduce learners to new content.
